# DSTYK Enhances Chemoresistance in Triple-Negative Breast Cancer Cells

**DOI:** 10.3390/cells11010097

**Published:** 2021-12-29

**Authors:** Stella C. Ogbu, Samuel Rojas, John Weaver, Phillip R. Musich, Jinyu Zhang, Zhi Q. Yao, Yong Jiang

**Affiliations:** 1 Department of Biomedical Sciences, J. H. Quillen College of Medicine, East Tennessee State University, Johnson City, TN 37614, USA; ogbusc@etsu.edu (S.C.O.); rojass@etsu.edu (S.R.); weaverjw@etsu.edu (J.W.); musichp@etsu.edu (P.R.M.); 2 Division of Infectious, Inflammatory and Immunologic Diseases, Department of Internal Medicine, Quillen College of Medicine, ETSU, Johnson City, TN 37614, USA; zhangj2@etsu.edu (J.Z.); yao@etsu.edu (Z.Q.Y.)

**Keywords:** breast cancer, DSTYK, CRISPR/Cas9, chemoresistance, TNBC

## Abstract

Breast cancer, as the most prevalent cancer in women, is responsible for more than 15% of new cancer cases and about 6.9% of all cancer-related death in the US. A major cause of therapeutic failure in breast cancer is the development of resistance to chemotherapy, especially for triple-negative breast cancer (TNBC). Therefore, how to overcome chemoresistance is the major challenge to improve the life expectancy of breast cancer patients. Our studies demonstrate that TNBC cells surviving the chronic treatment of chemotherapeutic drugs show significantly higher expression of the dual serine/threonine and tyrosine protein kinase (DSTYK) than non-treated parental cells. In our in vitro cellular models, DSTYK knockout via the CRISPR/Cas9-mediated technique results in apoptotic cell death of chemoresistant cells upon drug treatment. Moreover, DSTYK knockout promotes chemotherapeutic drug-induced tumor cell death in an orthotopic mouse model. These findings suggest that DSTYK exerts an important and previously unknown role in promoting chemoresistance. Our studies provide fundamental insight into the role of DSTYK in chemoresistance in TNBC cells and lay the foundation for the development of new strategies targeting DSTYK for improving TNBC therapy.

## 1. Introduction

Chemoresistance is the main reason for the failure of anti-breast cancer chemotherapy [1,2]. Triple-negative breast cancer (TNBC), defined as estrogen receptor (ER) negative, progesterone receptor (PR) negative, and human epidermal growth factor receptor 2 (HER2) negative, is the most lethal subtype of breast cancer due to its highly-aggressive characteristics, heterogeneity, and the availability of few treatment methods [3,4]. Currently, chemotherapy remains a standard neoadjuvant treatment of TNBC [5,6]. Unfortunately, chemoresistance is the most common treatment failure in TNBC patients [7].

Numerous mechanisms have been reported to explain the chemoresistance in TNBC cells including autophagy induction, unpredicted drug efflux, drug target shift, drug loss-of-function, cell death inhibition, epigenetic modifications, epithelial-to-mesenchymal transition (EMT), increased DNA damage repair, and cancer stem cells (CSCs) [8,9,10,11,12,13,14,15,16,17,18]. Recent studies to characterize TNBC tumors relying on the profiling of their transcriptome, proteome, epigenome, genome, and immuno-microenvironment significantly improved our understanding of the molecular heterogeneity of TNBC [17]. However, the discovery of effective and efficient treatment strategies is still an unmet clinical requirement. Therefore, it is urgent to find a strategy to overcome chemoresistance and save TNBC patients’ lives.

DSTYK, as a dual serine/threonine and tyrosine protein kinase, is expressed in multiple human tissues, including the brain, heart, kidney, lung, colon, and muscle [18]. Knockdown of DSTYK in zebrafish resulted in developmental defects in the spine, and further mechanistic studies suggested that DSTYK has involved in the fibroblast growth factor (FGF) signaling pathway by increasing the phosphorylation of extracellular-signal-regulated kinase (ERK) signaling in zebrafish [19]. In addition, DSTYK mutation was shown to upregulate the expression of two metastasis-related molecules, MMP2 and MMP9, in HPC3 cells by activating ERK1/2 signaling [19]. Furthermore, our group found that DSTYK promotes metastasis and chemoresistance via EMT in colorectal cancer [20]. To date, there is no available inhibitor targeting DSTYK for chemotherapy.

One popular chemotherapeutic drug combination, doxorubicin (DOX) plus docetaxel (DXL), for TNBC treatment has shown significant therapeutic efficacy in breast cancer therapy and has been frequently employed in clinical trials [21,22,23,24]. In this study, we employed the DOX and DXL combination (DOX/DXL) to select chemoresistant cells from two triple-negative breast cancer cell lines, SUM102PT and MDA-MB-468 cells. The cells surviving after DOX/DXL combination treatment show significant characteristics of CSCs and chemoresistance. Previous reports found that DSTYK promotes chemoresistance in colorectal cancer cells [20] and inhibits skin cell apoptosis [25]. Here, we report that DSTYK knockout (DSTYK^KO^) significantly attenuates the chemoresistance in TNBC cells, making chemoresistant cells sensitive to chemotherapeutic drug treatment in both in vitro and in vivo models. Therefore, we identified DSTYK as a potential chemotherapeutic target in the treatment of TNBC.

## 2. Materials and Methods

### 2.1. Xenografts Experiments in Mice

All of the animal experimental procedures are consistent with our mice protocols that were approved by the Institutional Animal Care and Use Committee at East Tennessee State University (ETSU). NOD.CB17-Prkdcscid/J mice (SCID) FVB mice, 4–6 weeks old, were purchased from the Jackson Laboratory and are maintained at the ETSU animal facility under the specified pathogen-free conditions. In all of the experiments, female mice were randomly selected. In the xenograft mouse model, approximately 2 × 10^5^ cells were injected into the flanks of 7–8-week-old SCID mice. When the tumor volume reached around 50 mm^3^, mice received therapy by intraperitoneal (i.p.) injection of the chemotherapeutic agent (DOX/DXL). DOX/DXL was administered to mice 2 times/week until some tumors reach a volume of ~1000 mm^3^. The tumors were sized every 3 days using calipers and the volume was calculated using a standard formula: Width^2^ × length × 0.5. Then, mice were euthanized by carbon dioxide and subjected to necropsy and tumor collection. Final tumors were weighed.

### 2.2. Cell Culture and Reagents

SUM102PT cells (BioIVT, NY, USA) were cultured in Ham’s F12 media with 5% FBS, 0.1% BSA, 5 µg/mL insulin, 1 µg/mL of hydrocortisone, 5 mM ethanolamine, 10 mM HEPES, 5 µg/mL apo-transferrin, 10 nM of triiodothyronine, 50 nM sodium selenite, and 10 ng/mL EGF. MDA-MB-468 cells (ATCC) were cultured in a DMEM medium containing 10% FBS and 1% antibiotic and antimycotic cocktail (Millipore, MO, USA). The cells were maintained at 37 °C in 95% air and 5% CO_2_ for all of the experiments.

### 2.3. Annexin V-FITC/7-AAD Double-Staining Assay

Cells were seeded in 12-well plates and incubated overnight for attachment, then the media was replaced with new media for the drug treatment. After the treatment, all of the floating and adherent cells were collected by trypsinization and centrifugation, followed by washing two times in phosphate-buffered saline (1 × PBS) before staining with Annexin V-FITC and 7-AAD, according to the manufacturer’s protocol (Cell Signaling Technology). Briefly, cells were resuspended in 500 µL 1× binding buffer containing 5 µL Annexin V-fluorescein isothiocyanate (FITC) and 10 µL 7-AAD in the dark for 15 min. For 7-ADD compensation, cells were incubated in a 70 °C water bath for 30 min. Then, we mixed live and dead cells, and thereafter, stained them with 7-ADD for compensation [26]. We used unstained cells as a negative control. For Annexin V compensation, we treated MDA-MB-468 cells with 0.5 and 5 µM doxorubicin for 24 h. Then, cells were stained with Annexin V [27]. The stained cells were analyzed with a flow cytometer, and the data were analyzed using FlowJo cell software (FlowJo, OR, USA) to measure apoptotic cells, live cells, and dead cells. Early apoptosis was designated as annexin V positive/7-AAD negative and late apoptosis was designated as annexin V positive/7-AAD positive. Necrosis was defined as annexin V negative/7-AAD positive. 

### 2.4. MTT Assay

The MTT assay was performed as previously described [28]. Briefly, cells were seeded in a 96-well dish with 200 μL media and incubated in a 37 °C and 5% CO_2_ incubator. After 24 h, various concentrations of DOX/DXL or control vehicle were added. Following the treatment for 48 h, cell viability was assessed by the MTT (3-[4,5-dimethylthiazol-2-yl]-2,5-diphenyl tetrazolium bromide; Sigma-Aldrich, St. Louis, MO, USA) assay, according to the manufacturer’s protocol. Viable fraction is expressed as the percentage of vehicle-treated control cells.

### 2.5. Western Blotting

Western blot (WB) analyses were performed as previously described [28]. Total proteins were separated by electrophoresis of a specified amount of whole cell lysate through 8–10% SDS-polyacrylamide gels, followed by transferring proteins to polyvinylidene difluoride (PVDF) membranes. Membranes were blocked with a 5% non-fat milk solution and incubated with primary antibodies for caspase-3, ERK, and p-ERK (Cell Signaling Biotechnology, Danvers, MA, USA), DSTYK (Santa Cruz Biotechnology, Dallas, TX, USA), GAPDH and Hsp90 (Santa Cruz Biotechnology). Secondary antibodies (Thermo Fisher Scientific, Waltham, MA, USA) conjugated to horseradish peroxidase and ECL (Bio-rad, Hercules, CA, USA) were used to detect signals, which were visualized by chemiluminescence. At least three independent experiments were performed for each WB assay.

### 2.6. Real Time-Quantitative PCR (RT-qPCR)

Total RNA was extracted using the RNeasy^®^Mini Kit (Qiagen, Germantown, MD, USA) and cDNA was synthesized from 1 μg of total RNA using High-Capacity cDNA Reverse Transcription Kits (Bio-Rad) plus SYB^®^ Gene Expression Assay Mix, sterile water, and Fast Universal PCR Master Mix (Applied Biosystems, Waltham, MA, USA) to measure the expression of specific genes. Gene expression levels were assessed by real-time RT-qPCR using the Taqman^®^ fast advanced master mix (Thermo Scientific, Waltham, MA, USA) and the CFX96 RT-PCR Detection System (Bio-Rad Laboratories Inc., Hercules, CA, USA). The PCR primer sequences used are: DSTYK forward primer: GAAGAGAAGTACCTCCAGC; DSTYK reverse primer: CAAGAAATCATTCACCAAGT. β-actin forward primer: CACCATTGGCAATGAGCGGTTC; β-actin reserve primer: AGGTCTTTGCGGATGTCCACGT. Gene expression was calculated and presented as fold changes.

### 2.7. Terminal Deoxynucleotidyl Transferase-Mediated dUTP Nick End-Labeling (TUNEL) Assay

The TUNEL assay was performed as described previously [20], following the supplier’s protocol, ABP Biosciences (catalog number: A050). Briefly, cells were cultured on coverslips in 24-well plates and fixed in 4% paraformaldehyde in PBS. The TdT reaction was performed for 1 h at 37 °C in a humidified chamber. Finally, the cells were stained with 4, 6-diamidino-2-phenylindole (DAPI) for 5 min. The fluorescence signal was examined and photos were taken with a Leica fluorescence microscope.

### 2.8. DSTYK Knockout (DSTYK^KO^) via Crispr/Cas9

Cells were transfected with DSTYK Crispr/Cas9 Knockout (KO) Plasmid (Santa Cruz, sc-404521), and HDR plasmids (Santa Cruz, sc-404521-HDR) using lipofectamine 3000 (Thermo Fisher Scientific), according to the manufacturer’s instructions. 48 h after transfection, cells were cultured in puromycin-containing media for the selection and cloning of DSTYK knockout (DSTYK^KO^) cells. Successful DSTYK^KO^ cells were confirmed by WB analysis.

### 2.9. Immunohistochemistry

Tumors derived from parent cells and resistant cells were collected following our previous immunohistochemistry protocol [20]. Paraffin-embedded tissue sections (5 µm thick) were incubated with an anti-DSTYK antibody overnight at 4 °C. The signals were detected using the Vectastain Elite ABC kit (Vector Laboratories, Burlingame, CA, USA), following the manufacturer’s protocol. Hematoxylin was used for counterstaining. The signal intensity was scored using the following scale: 0 (negative), 1 (weak), 2 (moderate), 3 (strong), 4 (middle strong), 5 (very strong). The staining was considered to be positive if the sum of distribution and intensity scores was greater than 1.

### 2.10. Statistical Analysis

Data was analyzed using Prism 7 software and is expressed as the mean ± S.D. Comparisons among multiple groups were made using a one-way ANOVA at a 95% confidence level (Turkey’s honest significance test). Comparisons between two groups were made using a parametric paired *t*-test for normally distributed data or non-parametric Wilcoxon paired *t*-test for non-normal distributions. Mice and sample groups were *n* > 3, unless otherwise indicated. Data was analyzed using ANOVA or the unpaired student *t*-test, and differences were considered significant at *p*-values of <0.05 (*), <0.01 (**), or <0.001 (***). Survival curves were generated using GraphPad Prism software.

## 3. Results

### 3.1. The Expression of DSTYK Is Related to the Survival Probability of TNBC Patients

**Figure 1:**
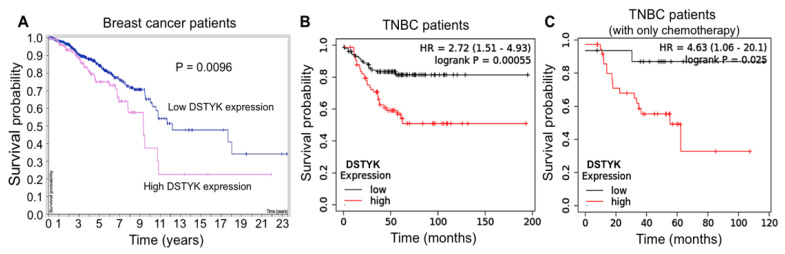
Statistical analysis of DSTYK-related survival probability in breast cancer patients. (**A**) Survival curves of breast cancer patients with differential DSTYK expression published in The Human Protein Atlas (HPA). Patients include all four cancer stages and those stages are not available (n/a). The survival probabilities were generated and are publicly available at website (www.proteinatlas.org, accessed on 14 December 2021). (**B**) Survival curves of TNBC patients and (**C**) TNBC patients only treated with chemotherapy from the Kaplan-Meier Plotter dataset statistical analysis (https://kmplot.com/analysis/index.php?p=service&cancer=breast, accessed on 14 December 2021).

It has been reported that DSTYK plays a predominant role in suppressing caspase-dependent apoptosis in some dermal cell types exposed to ultraviolet (UV) light [25], and we previously observed that DSTYK promotes metastasis and chemoresistance in colorectal cancer cells [20]. However, the role of DSTYK in the chemoresistance of TNBC cells has yet to be reported. Statistical analysis of the patient dataset indicates that a high DSTYK level is correlated with a high probability of death in breast cancer patients (Figure 1A, The Human Protein Atlas), TNBC patients (Figure 1B, Kaplan-Meier Plotter), and TNBC patients treated with chemotherapy only (Figure 1C, Kaplan-Meier Plotter). Furthermore, the image derived from NDEx v2.5.2 based on all of the available online databases provides more evidence for us to study DSTYK-related signaling pathways. DSTYK has been predicted to be involved in regulating apoptosis and cell death [25]. It may also participate in determining the TNBC cell fate by interacting with CDK13 [29], IKBKE [30], and/or TRIM25 [31], etc., (Supplemental Figure S1). These statistical data suggest that DSTYK may play a key role in chemoresistance in TNBC.

### 3.2. Establishment of Chemoresistant Cell Lines

**Figure 2:**
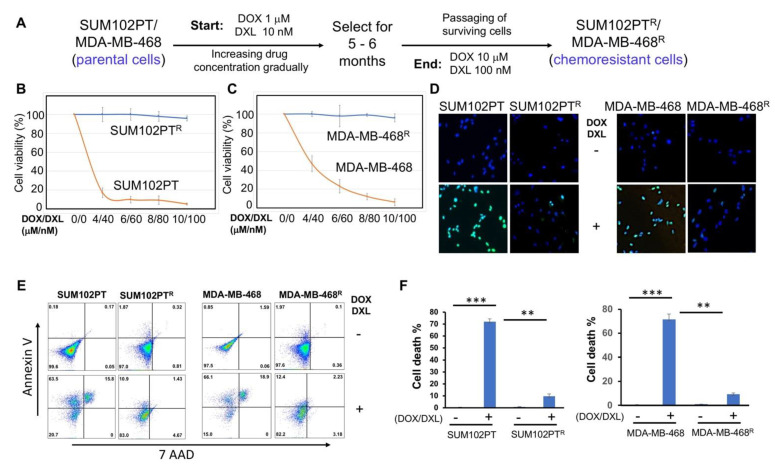
Schematic diagram of the procedure for generating chemoresistant TNBC cells. (**A**) SUM102PT and MDA-MB-468 cells were exposed to DOX/DXL. The concentration of drugs was gradually increased (DOX/DXL:1 µM/10 nM, 2 µM/20 nM, 4 µM/40 nM, 6 µM/60 nM, 8 µM/80 nM, 10 µM/100 nM) until stable proliferation at 10 µM/100 nM was established. (**B**) MTT assays to compare chemoresistance between SUM102PT and SUM102PT^R^ cells, and (**C**) between MDA-MB-468 and MDA-MB-468^R^ cells. The data presented represent the mean values ± SD (*n* = 3). (**D**) TUNEL apoptosis assays comparing the chemoresistance between SUM102PT and SUM102PT^R^, and between MDA-MB-468 and MDA-MB-468R cells. (**E**) Representative images from flow cytometry analyses to compare the levels of cell death by staining with cell surface apoptotic marker annexin V and the necrotic marker 7-ADD between parental cells and resistant SUM102PT cells treated with or without DOX/DXL (2 µM/20 nM) or MDA-MB-468 cells treated with or without DOX/DXL (4 µM/40 nM). (**F**) The quantification of the results from (**E**). The data represent mean values ± SD; ** *p* < 0.01; *** *p* < 0.001.

SUM102PT and MDA-MB-468 cells are two typical TNBC cell lines. The SUM102PT cell line was derived from a patient who had ductal carcinoma in situ (DCIS) with microinvasion and is considered as the only breast cancer cell line derived from this early stage TNBC [32]. MDA-MB-468 cells were extracted from a pleural effusion of the mammary gland and breast tissues, and it is commonly used to study TNBC metastasis, migration, and proliferation. These two cell lines were treated with DOX/DXL combination starting with a low concentration (Figure 2A). Dead cells were washed out, and then the surviving cells were collected and cultured in the complete culture medium absence of any drug. Thereafter, these cells were treated again with DOX/DXL at a higher concentration. The same selection was repeated for 5–6 months. Eventually, we acquired resistant SUM102PT^R^ and MDA-MB-468^R^ cells that survived in the presence of 10 µM DOX and 100 nM DXL (Figure 2A). The higher drug concentrations did not significantly induce cell death. We confirmed that the combination of DOX and DXL failed to induce significant cells death among the drug-resistant cell population using the MTT cell survival assay (Figure 2B,C) and the TUNEL assay that stains apoptotic cells (Figure 2D). Moreover, flow cytometry analyses were performed to detect cell surface apoptotic marker annexin V and the loss of plasma membrane integrity (uptake of 7-ADD) [28]. Our results showed that after the DOX/DXL treatment, there were no significant increases in annexin-V and 7-AAD signals in chemoresistant cells, whereas the majority of parental cells showed strong annexin V and 7AAD signals indicative of significant cell death (Figure 2E,F). These results indicate that SUM102PT^R^ and MDA-MB-468^R^ cell lines are resistant to the chemotherapeutic drug treatment and are optimum cell models for studying chemoresistance in TNBC cells.

### 3.3. DSTYK Expression Is Upregulated in Chemoresistant Cells

**Figure 3:**
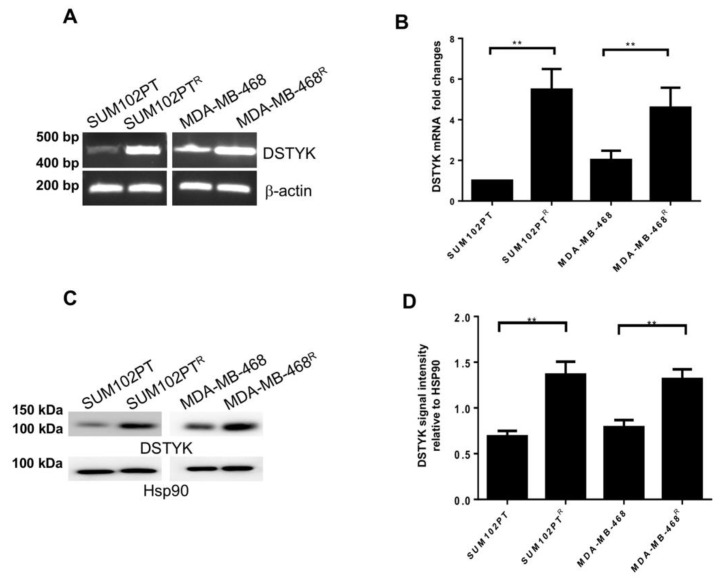
DSTYK expression correlates with chemoresistance. (**A**) The mRNA level of DSTYK between parental and chemoresistant cells by reverse-transcription PCR (RT-PCR) and (**B**) qPCR. (**C**,**D**) WB and quantification to detect the expressions of DSTYK in SUM102PT and SUM102PT^R^ cells, and MDA-MB-468 and MDA-MB-468^R^ cells, respectively. Hsp90 was used as the loading control. Experiments were repeated three times; ** *p* < 0.01; one-way ANOVA.

Furthermore, since previous findings that DSTYK is involved in cell apoptosis and chemoresistance in colorectal cancer cells [20], we planned to determine whether DSTYK also plays a role in the chemoresistance in SUM102PT^R^ and MDA-MB-468^R^ cells. First, we performed RT-PCR and qPCR analyses and the results indicate that the mRNA expression of DSTYK is considerably higher in chemoresistant cells than in parental cells for both SUM102PT and MDA-MB-468 cell lines (Figure 3A,B).

To compare the protein levels of DSTYK between SUM102PT and SUM102PT^R^ cells, and between MDA-MB-468 and MDA-MB-468^R^ cells, we performed immunoblotting assays. Our results demonstrate that DSTYK protein levels in chemoresistant cells are significantly higher than in parental cells (Figure 3C,D). Together, these results indicate that DSTYK is significantly upregulated in chemoresistant cells and suggest that DSTYK may play a role in chemoresistance in SUM102PT^R^ and MDA-MB-468^R^ cells.

### 3.4. DSTYK^KO^ Attenuates Chemoresistance in Chemoresistant Cells

**Figure 4:**
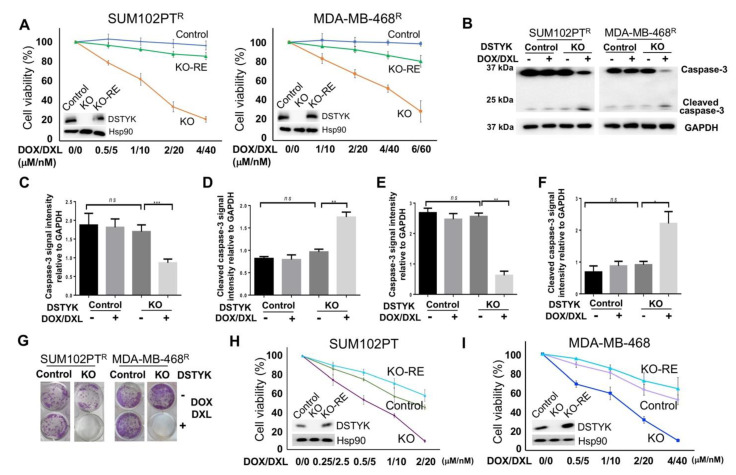
DSTYK is essential to the chemoresistance in TNBC cells. (**A**) MTT assays to compare the chemoresistance treated with different concentrations of DOX/DXL. The data presented represents the mean values ± SD (*n* = 3). The levels of cellular DSTYK protein are indicated by the included WB. Hsp90 was used as the loading control. (**B**) WBs to detect the apoptosis marker cleaved-caspase 3 before and after DOX/DXL treatment (0.5/5 μM/nM for SUM102PT^R^ cells; 1/10 μM/nM for MDA-MB-468^R^ cells). GAPDH was used as the loading control. (**C**–**F**) Quantification analyses of (**B**). (**G**) Clonogenic assays to confirm that chemoresistant cells regain chemosensitivity after DSTYK^KO^. DOX/DXL 4 μM/40 nm for SUM102PT^R^ cells and 6 μM/60 nm for MDA-MB-468^R^ cells. (**H**,**I**) MTT assays to compare the chemoresistance among parental control cells, parental DSTYK^KO^ cells, and parental DSTYK^KO-RE^ cells. Experiments were repeated three times; ns, not significant; * *p* < 0.05; **: *p* < 0.01; *** *p* < 0.001, one-way ANOVA.

To test whether DSTYK is essential for chemoresistance, we not only employed the Crispr/Cas9 technique to stably knock out DSTYK (DSTYK^KO^) expression in both SUM102PT^R^ and MDA-MB-468^R^ cells, but also restored DSTYK expression in KO (DSTYK^KO-RE^) cells. Then, we performed MTT assays to compare the chemotherapeutic response between control, DSTYK^KO^, and DSTYK^KO-RE^ chemoresistant cells (Figure 4A). The results reveal that DSTYK^KO^ chemoresistant cells showed dramatic cell death after the drug treatment, but the cell death was rescued in DSTYK^KO-RE^ chemoresistant cells. Furthermore, WB analyses indicate that the apoptosis marker cleaved-caspase 3 is significantly induced by the DOX/DXL combination treatment in the DSTYK^KO^ chemoresistant cells, whereas in control chemoresistant cells, cleaved-caspase 3 is barely induced. We did not observe any significant difference in the basal level of cleaved-caspase 3 between control chemoresistant and DSTYK^KO^ chemoresistant cells, which suggests that DSTYK^KO^ *per se* does not lower the viability of cells (Figure 4B–F). Clonogenic assays confirmed that much more apoptotic events occur in DSTYK^KO^ cells upon DOX/DXL treatment than in control cells (Figure 4G). Similarly, we knocked out DSTYK expression via Crispr/Cas9 in parental SUM102PT and MDA-MB-468 cells, followed by MTT assays to determine whether DSTYK^KO^ will cause more chemosensitivity. The results clearly demonstrate that more cell death is induced by DOX/DXL treatment in parental DSTYK^KO^ cells than in parental control cells in both SUM102PT and MDA-MB-468 cells (Figure 4H,I). In addition, DSTYK^KO^ does not lower the growth rate of cells before the drug treatment (Supplementary Figure S3), which further supports that DSTYK provides protection against chemotherapeutic drug-induced cell death. Moreover, there is no significant differences for cell death between DSTYK ^KO-RE^ and control parental cells (Figure 4H,I). Furthermore, we measured the phosphorylated ERK in control, DSTYK^KO^, and DSTYK^RE-KO^ cells. We found that DSTYK upregulates ERK phosphorylation, as shown in Supplemental Figure S2. All of these data indicate that DSTYK plays a key role in chemoresistance through inhibiting chemotherapeutic drug-induced cell death.

### 3.5. DSTYK Plays a Key Role in Chemoresistance through Inhibiting Apoptosis

**Figure 5:**
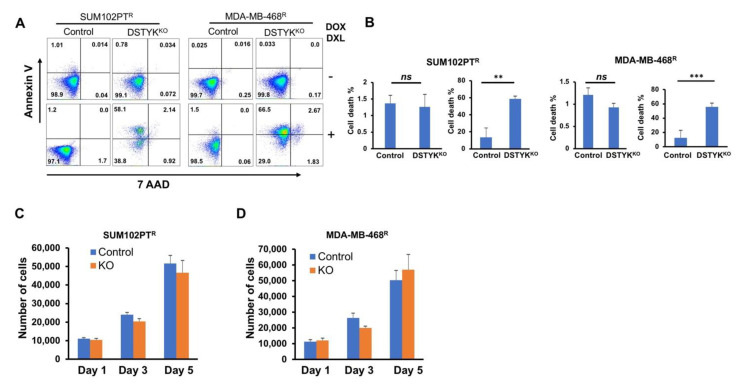
DSTYK^KO^ sensitizes chemoresistant cells to chemotherapy drug treatment. (**A**) Representative images from flow cytometry analyses to compare the levels of cell surface apoptotic marker annexin V and the necrotic marker 7-ADD between control and DSTYK^KO^ groups in SUM102PT^R^ cells or MDA-MB-468^R^ cells with or without DOX/DXL. DOX/DXL 2 μM/20 nm for SUM102PT^R^ cells and 4 μM/40 nm for MDA-MB-468^R^ cells. (**B**) The data in (**A**) are quantified. For each cell line, the left panel is the result for non-drug treated and the right panel is the result for drug-treated. The data represents mean values ± SD; *ns*, not significant; ** *p* < 0.01; *** *p* < 0.001. (**C**,**D**) DSTYK^KO^ resistant cell growth for 5 days and total numbers of cells were counted on days 1, 3, and 5 (*n* = 3 independent experiments) in SUM102PT^R^ cells and MDA-MB-468^R^ cells.

Flow cytometry analysis was performed to detect cell surface apoptotic marker annexin V and 7-ADD [28]. Our results show that after DOX/DXL treatment, there were few annexin V-positive control chemoresistant cells, whereas the majority of DSTYK^KO^ chemoresistant cells showed strong annexin V signals indicative of apoptosis (Figure 5A, B). To confirm, DSTYK plays a key role in chemoresistance by inhibiting apoptosis, we also performed flow cytometry analysis in DSTYK^KO-RE^ chemoresistant cells, our results showed that after DOX/DXL treatment, there were few annexin V-positive DSTYK^KO-RE^ chemoresistant cells that confirms restoring DSTYK expression rescued DSTYK^KO^-induced apoptosis after DOX/DXL treatment in chemoresistant cells (Supplemental Figure S4). Furthermore, in the DSTYK^KO^ chemoresistant cells, cells proliferations are not downregulated by DSTYK^KO^ per se (Figure 5C,D). These results indicate that DSTYK^KO^ sensitizes these cells to chemotherapeutic drug treatment, suggesting that DSTYK plays a key role in chemoresistance by inhibiting the apoptosis signaling pathway in TNBC.

### 3.6. Knockout of DSTYK Facilitates Tumor Regression during Drug Treatment in an In Vivo Orthotopic Mouse Model

**Figure 6:**
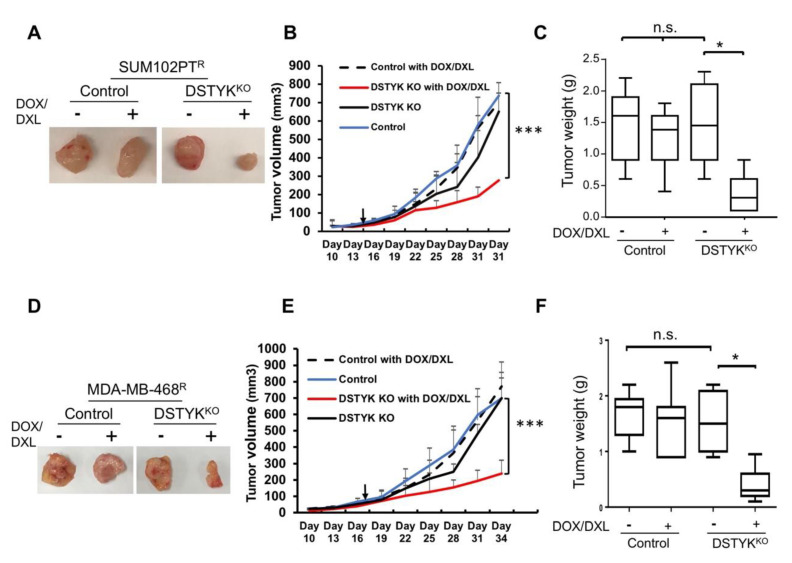
DSTYK promotes chemoresistance *in vivo*. (**A**) Representative images of one set of tumors derived from control and DSTYK^KO^ SUM102PT^R^ cells. (**B**) The graph represents the tumor growth volume observed for the different groups (as labeled) derived from SUM102PT^R^ cells. The data represents mean values ± SD. (**C**) Quantification of tumors after excision and being weighed at the end point; *n* = 7; n.s., not significant; * *p* < 0.05; *** *p* < 0.001, one-way ANOVA. (**D**) Representative images of one set of tumors derived from control and DSTYK^KO^ MDA-MB-468^R^ cells. (**E**) The graph represents the tumor growth volume observed for the different groups derived from MDA-MB-468^R^ cells (as labeled) until reaching the endpoint. The data represents mean values ± SD. (**F**) Quantification of tumors after excision and weighing at the end point; *n* = 7; n.s., not significant; * *p* < 0.05; *** *p* < 0.001, one-way ANOVA.

We employed an orthotopic mouse model to provide *in vivo* evidence that DSTYK^KO^ can attenuate chemoresistance. Approximately 2 × 10^5^ SUM102PT^R^ control or DSTYK^KO^ cells were subcutaneously implanted into the 4th mammary fat pads of 7-week-old immunodeficient NOD/SCID FVB mice. In each mouse, the control cells were implanted into the right 4th mammary fat pad and the DSTYK^KO^ cells were implanted into the left 4th mammary fat pad. After 3–4 weeks, seven mice carrying two tumors with similar sizes on each side were selected. A combination of DOX (4–5 mg/kg)/DXL (1–2 mg/kg) was administrated intraperitoneally (i.p.) into the mice 2 times/week for 3–4 weeks. After DOX/DXL treatment, the tumors developed from DSTYK^KO^ cells regressed significantly when compared to those from control resistant cells (Figure 6A–C). In confirmation of these data, similar results were observed in the same experiments performed with MDA-MB-468^R^ cells (Figure 6D–F). Moreover, we also checked the tumor growth curve in control and DSTYK^KO^ parental cells with DOX/DXL treatment. We found that control tumors are more resistant to DOX/DXL treatment when compared with DSTYK^KO^ tumors (Supplemental Figure S5). IHC staining showed that DSTYK expression is significantly higher in chemoresistant tumors when compared with parental tumors, as shown in Supplemental Figure S6. These results indicate that DSTYK can attenuate the chemosensitivity of tumor cells by inhibiting chemotherapeutic drug-induced tumor cell death. Taken together, all of the above results support that DSTYK plays a pivotal role in the chemoresistance of TNBC cells, and targeting DSTYK in TNBC chemotherapy may significantly improve the treatment efficacy.

## 4. Discussion

TNBC is a very deadly type of breast cancer and, presently, there is no ideal target available for effective therapy [33]. Chemotherapy remains the primary choice for most TNBC patients [9,34]. Successful chemotherapeutic treatments can eliminate cancer by inducing cancer cells to undergo apoptotic cell death [35], and, ideally, apoptosis is induced by chemotherapeutic agents in all TNBC cells [36,37,38]. Unfortunately, most TNBC patients will eventually develop resistance to chemotherapy, evading drug-induced cell death (deregulated apoptosis) and allowing the tumors in most TNBC patients to relapse and advance into metastasis, which is the reason for more than 90% of TNBC patients death [39]. Therefore, targeting deregulated apoptosis is an attractive approach to TNBC therapy [40]. As such, it is crucial to find a target for overcoming TNBC chemoresistance.

To identify the potential target that is responsible for TNBC chemoresistance, we employed two typical TNBC cell lines, SUM102PT and MDA-MB-468 cells. We further mimicked the clinical treatment process and eventually selected their chemoresistant cell clones, SUM102PT^R^ and MDA-MB-468^R^ cells. Based on our previous research in colorectal cancer cells [20], we found that DSTYK is significantly upregulated in chemoresistant TNBC cells when compared to their corresponding parental cells. Moreover, we found that DSTYK knockout (DSTYK^KO^) significantly attenuates the chemoresistance and renders chemoresistant cells regain sensitivity to chemotherapeutic drug treatment.

Although DSTYK is expressed in various tissues [41], its biological function is still elusive. It has been reported that DSTYK plays a predominant role in regulating both caspase-dependent and -independent apoptosis [42], and the underlying mechanisms are still under investigation. DSTYK was reported to promote ERK activation, and ERK inhibition has been proposed to improve cancer therapy. However, ERK inhibition did not significantly improve the TNBC treatment [43]. We speculate that shifting from ERK inhibitors to DSTYK inhibitors may hold greater promise in improving the TNBC treatment, since DSTYK may regulate the functions of many downstream factors in addition to ERK. We observed that DSTYK promotes metastasis and chemoresistance in colorectal cancer cells [20]. Here, we further found that DSTYK also plays a pivotal role in inhibiting chemotherapeutic agent-induced cell death in TNBC cells with both cellular and orthotopic models. Our studies indicate that DSTYK is a novel target to attenuate chemoresistance due to its inhibitory effect on apoptosis signaling in TNBC cells. DSTYK inactivation will affect the functions of many targets that may also participate in regulating the systematic apoptotic or survival processes. Therefore, inhibiting DSTYK activity will have a greater effect on TNBC treatment than inhibiting ERK activity, which will significantly impact on TNBC treatment regimen. Moreover, the large-scale statistical analyses of patient data support that a high DSTYK level is correlated with a high probability of death in breast cancer patients, TNBC patients, and TNBC patients treated with chemotherapy only.

It has been reported that the B-RafV600E inhibitor dabrafenib selectively inhibits the activity of receptor-interacting protein kinase 3 over receptor-interacting protein kinase 1, receptor-interacting protein kinase 2, and DSTYK. However, the inhibitory effect of dabrafenib on DSTYK was not clearly described [44]. To date, no specific inhibitors directly targeting DSTYK have been reported due to its novel role in the chemoresistance of breast cancer. We cannot exclude other kinase inhibitors, such as CDKs inhibitor purvalanol B, staurosporine, K-252a, and SB218078 (CHK1 inhibitor), which are published in “guidetopharmacology.org”, may affect DSTYK kinase activity, such as to execute indirectly more or less an inhibitory effect on DSTYK, but their biological functions are complicated since they target different kinases. Therefore, it is urgent to design a novel inhibitor specifically targeting DSTYK. To identify the mechanism of DSTYK in TNBC, we also predicted DSTYK signaling pathways in cancers using NDEx v2.5.2, the Network Data Exchange (NDEx) online data commons (www.ndexbio.org, accessed on 14 December 2021). This provides the direction for us to study DSTYK in clinical research. Since the next generation sequencing (NGS) is frequently employed in medical research, it may be necessary to perform more detailed expression profiling of primary TNBC specimens and their corresponding chemoresistant recurred specimens to identify more potential druggable targets including DSTYK, in order to achieve the success in improving the TNBC treatment.

## 5. Conclusions

Overall, our findings will significantly advance the medical strategy to the current dilemma of TNBC chemotherapy [45,46]. Our discovery will present DSTYK as a promising druggable target to facilitate the elimination of chemoresistant TNBC cells within TNBC.

## Data Availability

Data available on request.

## Supplementary Materials

The following figures are available online at https://www.mdpi.com/article/10.3390/cells11010097/s1, Figure S1: The predicted DSTYK-involved signaling pathway; Figure S2: DSTYK restoration rescues the chemoresistant phenotype; Figure S3: Cell growth rate comparison between control parental cells and DSTYK^KO^ parental cells; Figure S4: DSTYK^KO-RE^ rescues cell death of chemoresistant cells during chemotherapeutic drug treatment; Figure S5: The in vivo tumor growth and drug sensitivity of TNBC are dependent on DSTYK expression; Figure S6: DSTYK plays a key role in chemoresistant TNBC.
