# Supplementary material for: DSTYK Enhances Chemoresistance in Triple-Negative Breast Cancer Cells
Source: Cells. 2021 Dec 29;11(1):97. doi: 10.3390/cells11010097 (PMC8750327; doi:10.3390/cells11010097)
Supplement: Supplementary file 1 [file cells-11-00097-s001.zip › cells-1418992-supplementary.pdf]

cells MDPI

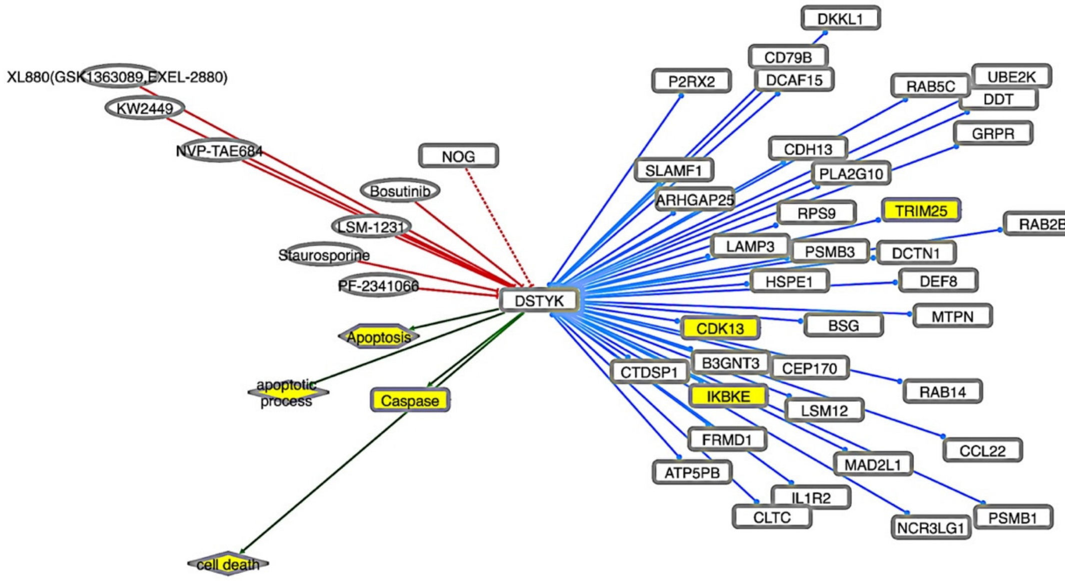

Figure S1. The predicted DSTYK-involved signaling pathway. The DSTYK signaling pathways are generated using NDEx v2.5.2, the Network Data Exchange (NDEx) online data commons (www.ndexbio.org), which can be used to improve the quality and abundance of biological networks relevant to the cancer research community. The yellow color indicates that DSTYK is highly relative to the molecules which have been reported to have important functions in cancer.

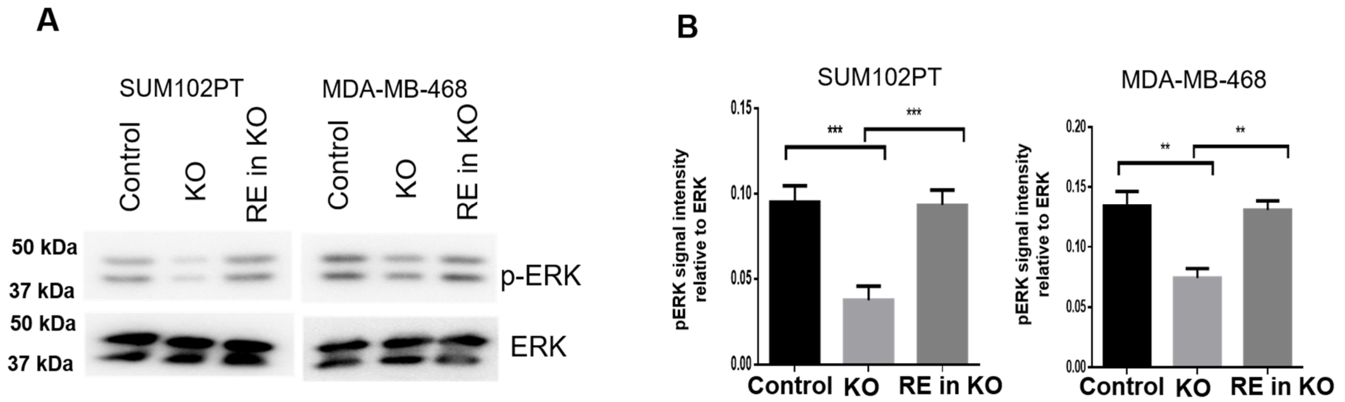

Figure S2. DSTYK restoration rescues the chemoresistant phenotype. (**A**) DSTYK influences the level of phosphorylated ERK. WBs to detect phosphorylated ERK and total ERK expressions. (**B**) The quantification of WB results from (**A**). The data presented represent the mean values ± SD (n = 3). Experiments were repeated three times. ** $p < 0.01$; *** $p < 0.001$.

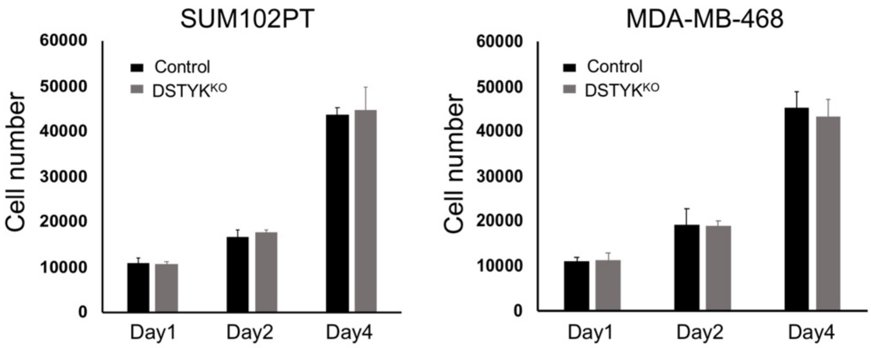

Figure S3. Cell growth rate comparison between control parental cells and DSTYK^{KO} parental cells. The data represent mean values ± SD (n = 3) in SUM102PT and MDA-MB-468 cells.

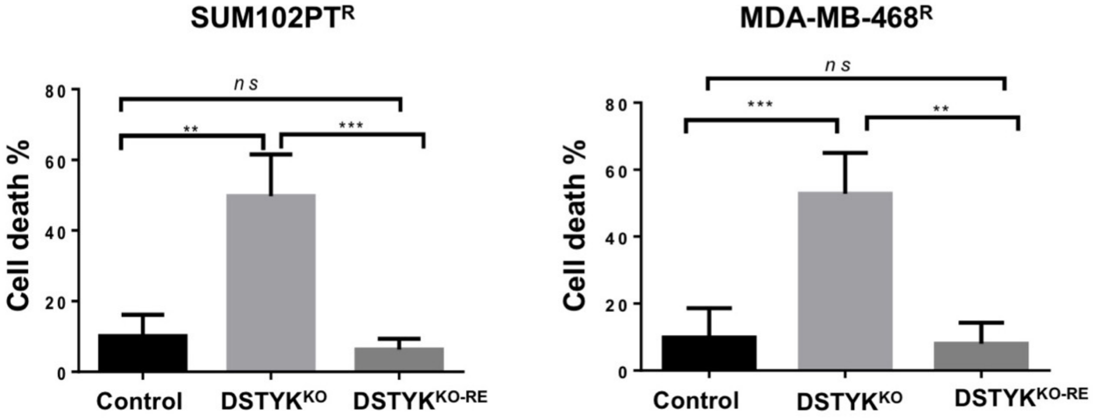

Figure S4. DSTYK^{KO-RE} rescues cell death of chemoresistant cells during chemotherapeutic drug treatment. Cells were stained with Annexin V and 7-ADD and analyzed by flow cytometry. The results are quantified from control, DSTYK^{KO}, and DSTYK^{KO-OE} cells. Experiments were repeated three times. ns, not significant; ** $p < 0.01$; *** $p < 0.001$, one-way ANOVA.

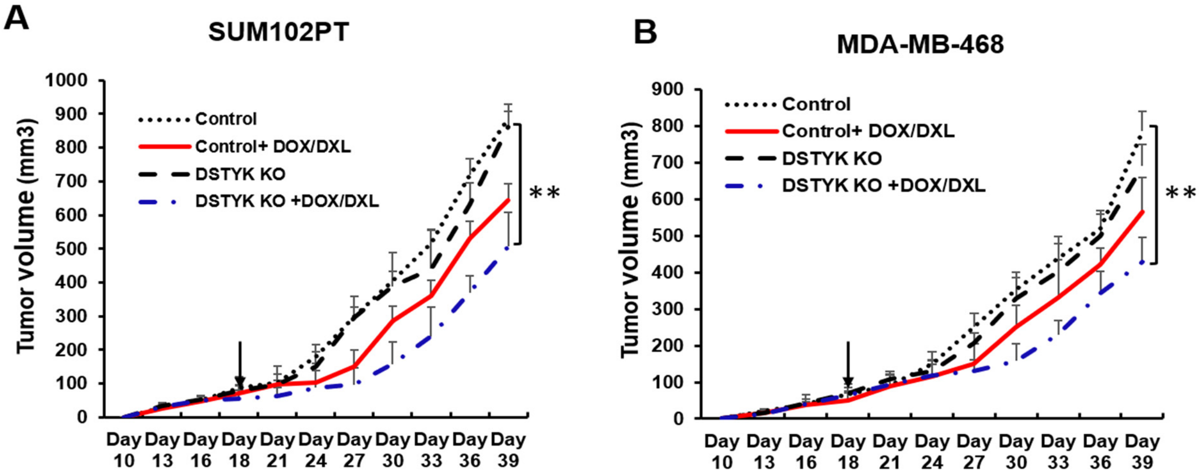

Figure S5. The in vivo tumor growth and drug sensitivity of TNBC are dependent on DSTYK expression. Using an orthotopic mouse model, tumors were induced with parental TNBC cells (control) or their DSTYK^{KO} derivatives in mammary fat pads with or without DOX/DXL treatment. (**A**) The tumor growth curve is measured in control and DSTYK^{KO} groups in SUM102PT cells. Tumor size was measured every 3 days and then recorded starting from the tumor growth became visible to the endpoint. (**B**) The tumor growth curve is measured in control and DSTYK^{KO} groups in MDA-

MB-468 cells. Tumor size was measured every 3 days and then recorded. ** $p < 0.01$, one-way ANOVA.

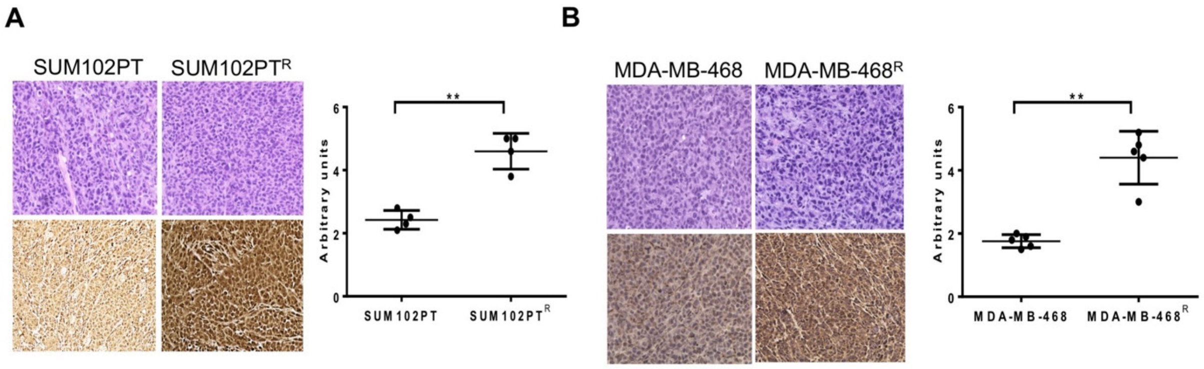

Figure S6. DSTYK plays a key role in chemoresistant TNBC. (**A**) DSTYK specific IHC staining of the tumors derived from parental and chemoresistant SUM102PT cells. (**B**) DSTYK specific IHC staining of the tumors derived from parental and chemoresistant MDA-MB-468 cells. Data are shown as mean ± SD (** $p < 0.01$)
